# Factors influencing medical students’ adoption of AI educational agents: an extended UTAUT model

**DOI:** 10.1186/s12909-025-08234-z

**Published:** 2025-12-05

**Authors:** Xiaoxiong Zhao, Wankun Liu, Sansi Yue, Jia Chen, Dan Xia, Kaijian Bing, Xiaotian Xia, Keshan Wang

**Affiliations:** 1 Faculty of Artificial Intelligence in Education, Central China Normal University, Wuhan, 430079 Hubei China; 2 Department of Surgical Education Research, Union Hospital, Tongji Medical College, Huazhong University of Science and Technology, 430022 Wuhan, China; 3 Department of Nuclear Medicine, Union Hospital, Tongji Medical College, Huazhong University of Science and Technology, No.1277 Jiefang Avenue, Wuhan, 430022 Hubei China; 4 Department of Urology, Tongji Medical College, Union Hospital, Huazhong University of Science and Technology, Wuhan, 430022 China

**Keywords:** Medical AI educational agents, UTAUT model, Medical students, PLS-SEM analysis, Medical education

## Abstract

**Background:**

Artificial intelligence (AI) is reshaping the landscape of medical education with unprecedented depth and breadth. As technologies like large language models and natural language processing advance, AI agents with multimodal interaction capabilities—such as intelligent teaching assistants and virtual simulation labs—are demonstrating immense potential. Concurrently, medical students face challenges including a disconnect between theoretical knowledge and clinical practice, excessive cognitive load, and a lack of personalized practical opportunities. Medical education AI agents are poised to address these issues, but their successful integration hinges on student acceptance and adoption. This study aims to fill a gap in the current empirical research by investigating the key psychological mechanisms and behavioral factors that influence medical students’ adoption of AI educational agents.

**Methods:**

This study constructed an extended Unified Theory of Acceptance and Use of Technology (UTAUT) model by integrating four key variables tailored to the medical education context: AI Trust, Perceived Risk, Hedonic Motivation, and Trialability. A cross-sectional survey was conducted with an initial sample of 200 clinical medicine students following their interaction with a custom-developed interactive medical education AI agent. After excluding invalid responses, a final valid sample of 155 participants was retained. Partial Least Squares Structural Equation Modeling (PLS-SEM) was employed to validate the theoretical model and test the research hypotheses.

**Results:**

The constructed model demonstrated strong explanatory power, successfully accounting for 85.3% of the variance in students’ behavioral intention (R² = 0.853). Effort Expectancy (β = 0.362, *p* < 0.001) and Performance Expectancy (β = 0.297, *p* < 0.001) were the strongest direct positive predictors of behavioral intention, with Facilitating Conditions (β = 0.258, *p* = 0.002) also showing a significant impact. A noteworthy finding was that Social Influence had no significant effect on behavioral intention (β = 0.038, *p* = 0.633). Furthermore, Hedonic Motivation had a significant positive influence on both Effort Expectancy (β = 0.818, *p* < 0.001) and Performance Expectancy (β = 0.237, *p* < 0.001). AI Trust, Trialability, and lower Perceived Risk also significantly enhanced students’ Performance Expectancy.

**Conclusions:**

The findings indicate that for medical students, who are highly autonomous professional learners, the intrinsic value of an AI educational tool (i.e., its utility and ease of use) is the dominant factor in their adoption decisions, far outweighing the social influence of peers or authorities. Therefore, the key to successfully promoting such technologies lies in building users’ intrinsic trust, reducing their perceived risks, and providing an engaging, immersive learning experience. These findings provide a solid empirical basis for the optimal design of medical AI educational agents and for strategies to effectively integrate them into the curriculum.

**Supplementary Information:**

The online version contains supplementary material available at 10.1186/s12909-025-08234-z.

## Introduction

### Background and problem statement

Breakthroughs in large language models, computer graphics, and natural language processing are enabling AI agents with multimodal interaction capabilities to demonstrate immense potential in medical education. These agents, characterized by their superior abilities in natural language understanding, memory, tool use, and decision-making, are manifesting as intelligent teaching assistants, virtual instructors, and simulated laboratories. Consequently, the paradigm of AI in medical education is rapidly shifting from “tool enablement” to “scenario reconstruction.” Through multimodal human-computer interaction, personalized virtual teaching scenarios, and the use of virtual mentors and patients, Medical AI educational Agents are reshaping traditional pedagogical models.

Medical education is distinguished by its high degree of professionalism and practical demands, requiring students not only to master a vast body of knowledge but also to develop profound clinical reasoning and robust professional skills. However, medical students currently face significant challenges, including dense curricula, heavy cognitive loads for knowledge comprehension, and a lack of sufficient hands-on training opportunities [1]. The application of medical AI educational agents offers a promising pathway to address these issues. By providing functions such as virtual instructor-led courses, simulated laboratories, and intelligent assistant Q&A sessions, these agents can create highly personalized and adaptive learning environments. Furthermore, they can offer students opportunities for simulated clinical consultations, real-time feedback on diagnostic reasoning, and on-demand access to the latest medical knowledge [2], effectively compensating for the shortcomings of traditional teaching methods.

However, the efficacy of these advanced tools depends not only on their technical capabilities but, more critically, on their acceptance and willingness to be used by future medical professionals [3]. A significant ‘adoption gap’ currently exists in their application: on one hand, AI technology is advancing rapidly in the medical field; on the other, medical students—as a cautious, risk-averse, and psychologically complex group—exhibit a considerable disparity between their willingness to adopt and their actual usage of these agents. Therefore, an in-depth investigation into the key factors influencing medical students’ adoption of AI educational agents is crucial to ensure these technologies genuinely enhance educational quality and clinical service standards.

Existing research has predominantly focused on the technical performance and educational effectiveness of AI agents [2, 4, 5], their functions in auxiliary medical diagnosis [6, 7], system design [8], and application overviews [9]. There remains a scarcity of empirical studies that apply established theoretical frameworks to systematically examine the psychological and experiential factors influencing adoption among the key user group of medical students. This is particularly true for medical AI educational agents, which function as “AI service providers.” Their role is fundamentally different from front-line clinical AI systems used for disease diagnosis or medical imaging analysis. The core function of an educational agent is to assist and augment students’ cognitive tasks, not to replace core clinical decision-making processes. This role difference is likely to elicit distinct psychological responses and acceptance patterns from users. This paper, therefore, aims to provide empirical evidence to bridge the gap between the potential of AI technology and its practical application in medical education.

### Theoretical framework and model extension

A medical student’s willingness to adopt and effectively use an AI agent is influenced by a variety of factors, including subjective perceptions, attitudes, and behavioral intentions. The Unified Theory of Acceptance and Use of Technology (UTAUT), proposed by Venkatesh et al. [10], provides a systematic theoretical framework for understanding technology adoption behavior and has been widely applied in the educational field [11, 12]to identify key variables affecting user acceptance of educational technologies. This study selects UTAUT as its foundational analytical framework. Although its predecessor, the Technology Acceptance Model (TAM), has been extensively used, its focus on perceived usefulness and ease of use is insufficient to fully capture the complex and highly socialized context of medical education. UTAUT offers a more robust starting point by incorporating key contextual determinants such as ‘Social Influence’ and ‘Facilitating Conditions’.

Given that medical AI educational agents are interactive, emergent technologies with a degree of autonomy, their application in a high-stakes field introduces psychological dimensions of trust, experience, and risk that are not fully covered by traditional models. Therefore, a core theoretical task of this research is to extend the UTAUT model to construct a more contextually explanatory framework. To achieve this, this study introduces four key external variables—AI Trust, Hedonic Motivation, Trialability, and Perceived Risk—to enhance the model’s applicability and explanatory power in the context of AI agent adoption.

First, the medical education AI agent acts not merely as a tool but as a pedagogical partner. This implies that the student-AI relationship transcends simple functional interaction and enters the realm of trust. Drawing on Social Presence Theory [13], which posits that a user’s perception of “others being present” in a mediated environment influences communication quality and trust-building, we argue that when an AI exhibits human-like social attributes, it can foster students’ emotional connection and trust [14, 15]. This interaction-generated AI Trust (AIT) serves as a profound, internalized complement to the ‘Social Influence (SI)’ construct in UTAUT, which focuses more on external normative pressures.

Second, effective medical learning is an active, practical process of cognitive construction, not passive knowledge acquisition. Grounded in Embodied Cognition Theory [16], which asserts that learning depends on the body’s interaction with the environment through operation, perception, and feedback [17], we recognize that the controllable and immersive embodied experiences offered by AI agents (e.g., in virtual consultations) can stimulate students’ enjoyment and intrinsic motivation. Consequently, Hedonic Motivation (HM)—the pleasure derived from the interaction itself [18]—and Trialability (TR)—the ease of experimenting with the technology [19]—become critical variables explaining why students are willing to “engage” and “explore”. These constructs supplement UTAUT’s utilitarian motives (e.g., Performance Expectancy) by explaining adoption from the perspective of the learning experience itself.

Finally, the high-stakes nature of the medical field necessitates the inclusion of Perceived Risk (PR). Perceived Risk theory [20] suggests that users evaluate potential uncertainty and negative consequences when adopting new technologies. In the context of medical education, erroneous advice from an agent could negatively shape a student’s clinical judgment, making perceived risk an indispensable psychological variable [21]. Future clinicians must weigh multiple potential risks, including ‘performance risk’ (forming incorrect diagnostic habits), ‘liability risk’ (unclear accountability for AI-assisted errors), and ‘cognitive risk’ (atrophy of clinical reasoning skills due to over-reliance). In this high-risk environment, Perceived Risk not only directly impacts the intention to use but also fundamentally shapes a student’s initial assessment of the tool’s value (i.e., Performance Expectancy).Research indicates that the influence of perceived risk on adoption intent may vary significantly across different healthcare roles, highlighting its critical and complex nature as a potential barrier to adoption [22, 23].

Based on this theoretical foundation, this study integrates AI Trust, Hedonic Motivation, Trialability, and Perceived Risk into the UTAUT framework to build a technology adoption model better suited to the unique context of medical education.

### Research hypotheses

#### Performance expectancy (PE)

Performance Expectancy refers to the degree to which a medical student believes that using an AI agent will help them attain gains in academic achievement, clinical skills, and learning efficiency [10, 24]. In the context of medical education, this means students believe the agent can help them better understand complex medical concepts, improve diagnostic accuracy, and efficiently access the latest medical knowledge. Previous research has consistently shown that performance expectancy is a significant positive predictor of behavioral intention [24, 25]. If medical students perceive that an AI agent can genuinely support their studies and future clinical work, their intention to use it will increase. Therefore, we hypothesize:

- *H1 *Performance Expectancy has a significant positive effect on medical students’ behavioral intention to use the AI agent.

#### Effort expectancy (EE)

Effort Expectancy is the degree of ease associated with the use of the AI agent, similar to the concept of “perceived ease of use” in TAM [10, 26, 27]. It encompasses aspects such as the user-friendliness of the interface, the simplicity of interaction flows, and the time and effort required to learn the system. When medical students find the agent easy to operate and master, they are more likely to use it. Numerous studies on educational technology have confirmed that effort expectancy has a significant positive effect on usage intention [28, 29]. Therefore, we hypothesize:

- *H2* Effort Expectancy has a significant positive effect on medical students’ behavioral intention to use the AI agent.

#### Social influence (SI)

Social Influence refers to the degree to which a medical student perceives that important others (e.g., instructors, peers, experts in the medical field) believe they should use the AI agent [10, 30]. In addition, Previous studies have demonstrated that users are more inclined to engage with a system when it is endorsed by significant figures in their social circle [31]. This positive correlation between social influence and technology adoption intention has also been observed in the context of generative AI [19, 32]. Therefore, we hypothesize:

- *H3 *Social Influence has a significant positive effect on medical students’ behavioral intention to use the AI agent.

#### Facilitating conditions (FC)

Facilitating Conditions refer to a medical student’s perception of the available organizational and technical infrastructure to support the use of the AI agent, including adequate device support, stable network environments, and technical assistance [10, 33, 34]. While the original UTAUT model posited that FC primarily influences actual use rather than intention, we argue that in the early stages of AI agent adoption, users often conduct an “Anticipatory Evaluation.” They assess not only the tool’s utility and ease of use but also whether they will have the necessary resources in the future. The expectation of inadequate support can lower adoption intention from the outset. This reasoning is supported by recent studies that have found a direct, significant effect of FC on behavioral intention in various AI-related contexts [33]. Therefore, we hypothesize:

- *H4* Facilitating Conditions have a significant positive effect on medical students’ behavioral intention to use the AI agent.

#### AI trust (AIT)

AI Trust is the student’s subjective belief in the reliability, accuracy, and security of the AI agent [35]. Given the highly specialized nature of the knowledge provided by the agent, students must trust its information and reasoning capabilities to consider it an effective learning tool. Research has shown that higher user trust in an AI system leads to a higher perception of its usefulness, or Performance Expectancy [14, 36]. In educational settings, trust has been empirically linked to a user’s perception of a system’s efficacy [37]. This relationship—where AI Trust influences behavioral intention through Performance Expectancy—has been empirically supported in previous studies. For instance, a study on Generation Z’s acceptance of online technology in Thailand validated the significant mediating paths of “Trust → Performance Expectancy → Behavioral Intention” and “Trust → Effort Expectancy → Behavioral Intention” using Structural Equation Modeling (SEM) and mediation analysis [38]. Specifically, their findings indicated that the impact of trust on behavioral intention was primarily indirect, mediated through both Performance Expectancy and Effort Expectancy. Similarly, the integrated model developed by Gefen et al. demonstrated that trust significantly enhances users’ Perceived Usefulness, which in turn influences their intention to use the technology [14].Therefore, we hypothesize:

- *H5* AI Trust has a significant positive effect on medical students’ Performance Expectancy.

#### Trialability (TR)

Trialability refers to the degree to which a medical student can experiment with the AI agent before full adoption [19, 39]. According to Rogers’ Diffusion of Innovations Theory [19], trialability is a core characteristic influencing adoption rates by reducing uncertainty and enhancing perceived usefulness (i.e., Performance Expectancy). If students can test the agent’s core functions (e.g., simulated diagnosis) in a low-stakes environment, they are more likely to build confidence in its educational benefits. This relationship has been validated in educational technology research [36, 37]. Therefore, we hypothesize:

- *H6* Trialability has a significant positive effect on medical students’ Performance Expectancy.

#### Perceived risk (PR)

Perceived Risk refers to the user’s perception of potential negative consequences associated with using the AI agent, such as receiving incorrect medical information, data privacy breaches, or system failures [37]. In the high-stakes context of medical education, perceived risk is expected to first diminish a student’s assessment of the AI system’s value (Performance Expectancy) before ultimately affecting their adoption intention [37]. Therefore, we hypothesize:

- *H7* Perceived Risk has a significant negative effect on medical students’ Performance Expectancy.

#### Hedonic motivation (HM)

Hedonic Motivation refers to the fun or pleasure derived from using the AI agent [40]. In medical education, where content is often complex and cognitively demanding, an engaging and enjoyable learning experience can stimulate student participation. This heightened engagement can make the system seem easier to use, thereby increasing Effort Expectancy, and also lead students to believe they are gaining more from the experience, thus enhancing Performance Expectancy. This logic is supported by empirical studies showing that hedonic motivation is a key predictor of technology adoption that significantly influences both performance and effort expectancies [37, 40, 41]. Therefore, we hypothesize:

- *H8 *Hedonic Motivation has a significant positive effect on medical students’ Performance Expectancy.
- *H9 *Hedonic Motivation has a significant positive effect on medical students’ Effort Expectancy.

### Research model

**Fig. 1:**
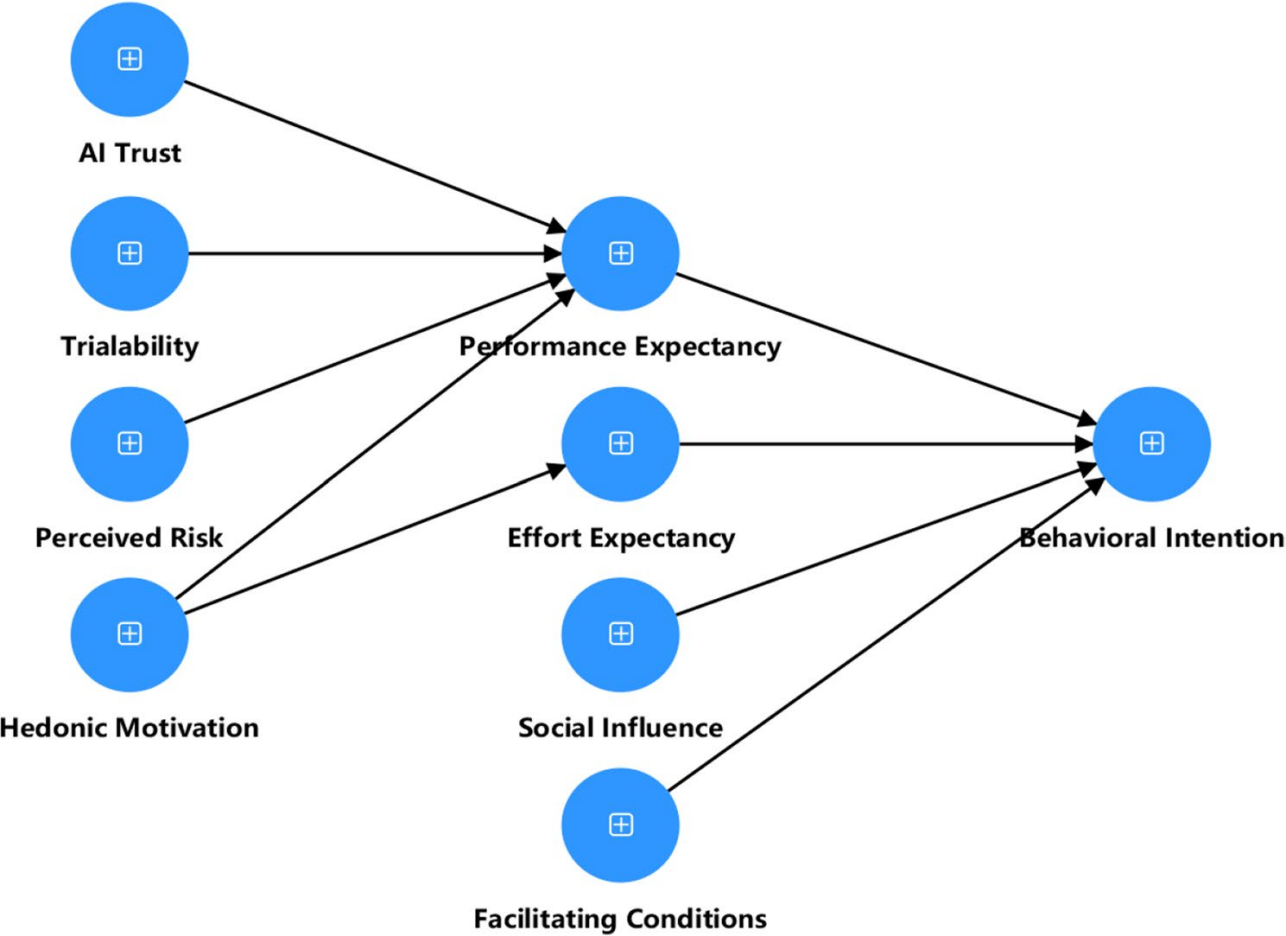
Model of the research

Figure 1 presents the research model for this study, which clearly depicts the relationships among AI Trust, Trialability, Perceived Risk, Hedonic Motivation, Performance Expectancy, Effort Expectancy, Social Influence, Facilitating Conditions, and Behavioral Intention. The model is designed to explore how these variables interact and influence the learning experiences and behavioral intentions of medical students during their use of the AI agent.

## Methods

### Study design

This study utilizes a cross-sectional survey design. This method is intended to systematically collect data at a specific point in time regarding medical students’ perceptions, attitudes, and behavioral intentions toward medical education intelligent agents. Through the quantitative analysis of this data, the study is able to test the relationships between the variables in the proposed extended UTAUT model and validate the relevant research hypotheses.

### Research instrument

The experimental tool used in this study was a custom-developed interactive medical education AI agent built on the Unity engine. The agent was designed to create a comprehensive learning environment for medical students, integrating knowledge acquisition, skills training, and contextual experience through the consolidation of multimodal teaching resources and advanced AI technologies.

The core of the agent integrates three key components:

*Large Language model (LLM)*: integration The agent utilizes an API to make real-time calls to the DeepSeek-R1 model, which serves as its cognitive and reasoning core, responsible for processing complex medical questions and generating conversational content.

*Voice interaction capability*: It incorporates iFlytek’s Automatic Speech Recognition (ASR) and Text-to-Speech (TTS) services to enable fluid, natural language voice interactions.

*Multimedia content generation and presentation*: The agent uses tools such as “YouYan” to generate digital human instructional videos and renders 360° panoramic scenes within the Unity engine to deliver an immersive virtual simulation experience.

Based on this architecture, three core functional modules were designed to support students’ online learning activities:

*Digital human video instruction* This module provides students with standardized instructional video courses delivered by an AI digital human, covering key theoretical medical knowledge. To ensure content quality and uniformity, these videos were automatically generated using a professional digital human video platform (“YouYan”) by inputting standardized teaching scripts, resulting in a digital instructor with realistic lip-syncing, facial expressions, and natural movements. This module was designed to enhance the enjoyment and appeal of the learning process through a vivid, anthropomorphic presentation, directly targeting the Hedonic Motivation (HM) construct in our research model.

*AI Assistant and Virtual Patient*: This module allows students to engage in real-time communication with the AI via text or voice. It can function as an omniscient AI teaching assistant to answer medical questions and provide learning materials, or it can instantly switch its “persona” to role-play as a “virtual patient” with a specific disease, facilitating highly realistic simulated consultations. The technical workflow is as follows: the user’s voice input is converted to text by the iFlytek ASR service. This text, along with a predefined persona prompt (e.g., “You are a professional AI medical teaching assistant” or “You are a patient with a specific disease”), is sent to the DeepSeek LLM API. The model generates a contextually appropriate response, which is then converted back into natural-sounding speech via the iFlytek TTS service. By providing instant feedback and natural language interaction, this module aims to lower the barrier to use, thereby enhancing Effort Expectancy (EE). Concurrently, the high-fidelity clinical simulations are intended to build student confidence in the system’s reliability, thus strengthening AI Trust (AIT).

*Immersive Simulated Scene*: This module is designed to enhance Trialability (TR) and Facilitating Conditions (FC) by providing an explorable virtual environment. Built using 360° panoramic images of real laboratories and clinical settings, the module is rendered with interactive functionalities in the Unity engine. Students can freely look around the scene. When they hover over or click on key objects (e.g., medical equipment), interactive hotspots trigger the display of high-resolution close-up images and detailed textual descriptions. This allows students to safely and intuitively familiarize themselves with the environment and equipment before any formal procedures. By enabling low-risk exploration, this module’s primary purpose is to improve the agent’s Trialability and provide the necessary environmental support corresponding to Facilitating Conditions.

**Fig. 2:**
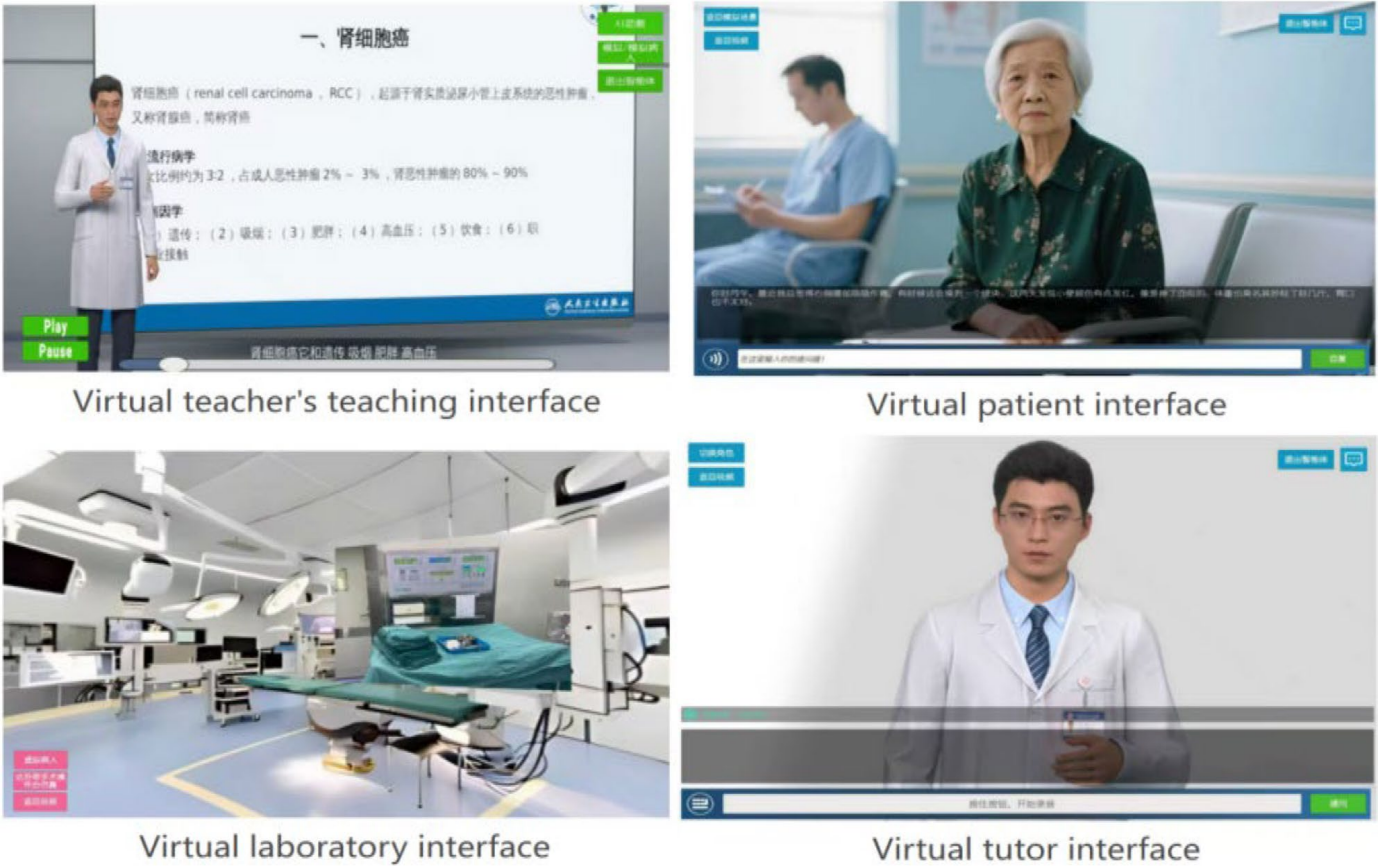
Screenshots of the core module interfaces of the medical education intelligent agent

**Fig. 3:**
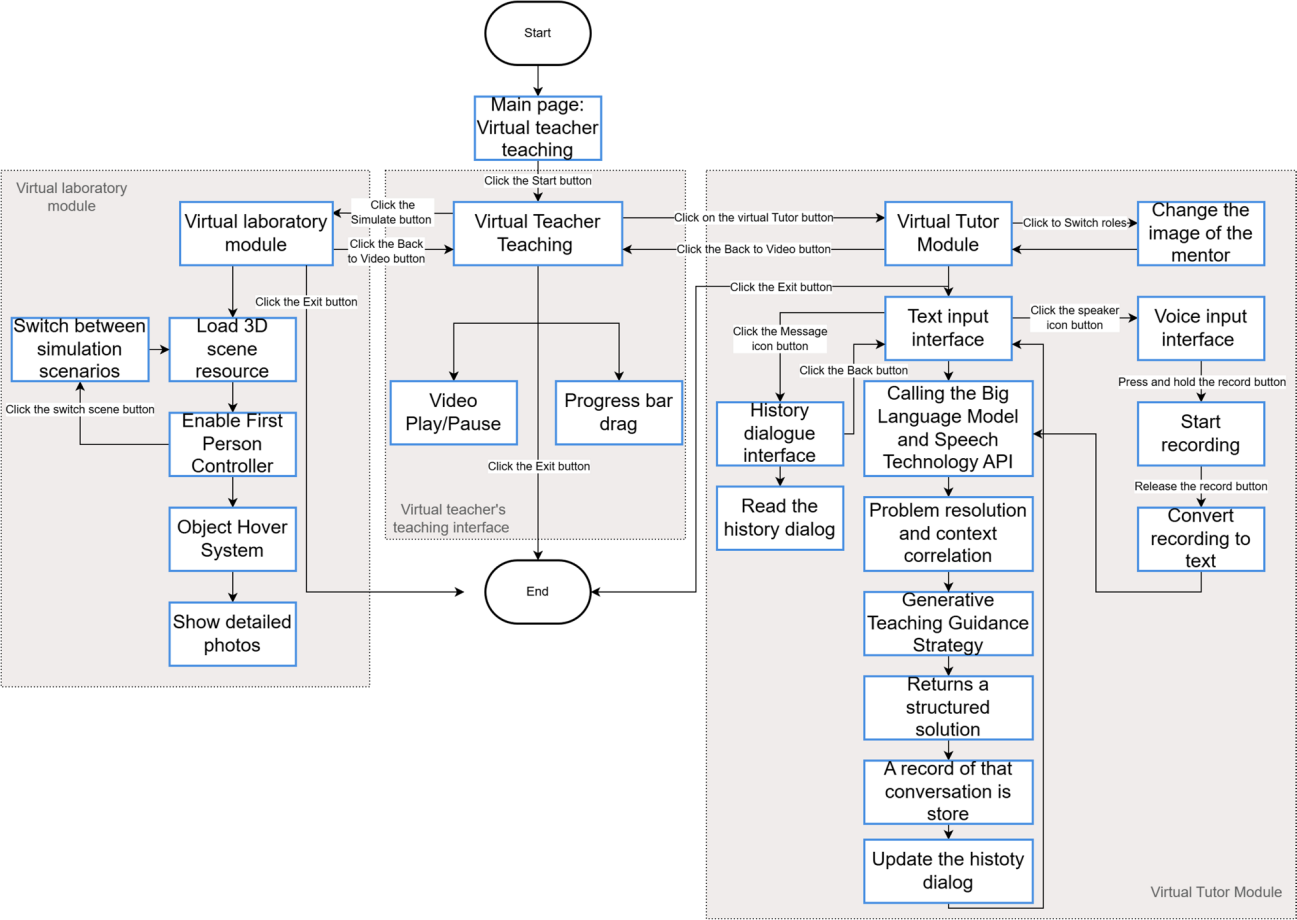
System flowchart of the medical education intelligent agent

To visually represent the functionalities and interaction methods of the research instrument, Fig. 2 displays screenshots of the agent’s core modules, and Fig. 3 illustrates the system’s workflow diagram.

### System performance and usage monitoring

To complement the self-reported survey data, backend operational metrics of the AI agent were collected during the interaction period from May to June, 2025.

Usage Data: Daily token consumption and API request counts were recorded through the system’s backend. Token consumption indicates the volume of AI-generated content accessed by users, while API request counts reflect the frequency of interactions with the large language model.

System Performance: Client-side monitoring recorded the average CPU usage during typical interaction sessions. The system consistently operated within a CPU usage range of approximately 14% to 16%, suggesting stable performance across user sessions (Supplementary 1).

### Data collection and sample

A total of 200 undergraduate students from Tongji Medical College, Huazhong University of Science and Technology, were initially recruited for this study. After excluding responses with excessively short completion times or missing data, 155 valid responses were retained for analysis. The demographic characteristics of the final sample are summarized in Table 1. Among the participants, 92 were female (59.4%) and 63 were male (40.6%), with the majority aged between 18 and 21 years. The sample included students from Year 1 (31.0%), Year 2 (29.0%), and Year 3 (40.0%), representing a wide range of academic stages and geographical backgrounds, thereby enhancing the diversity and representativeness of the sample. This demographic profile is broadly consistent with the overall enrollment structure of the institution’s clinical medicine program.

**Table 1:** Demographic characteristics of the initial recruited participants (*N* = 155)

Variable	Category	*N*	Percentage (%)
Gender	Male	63	40.6
	Female	92	59.4
Age	Under 18	6	3.9
	18–19 years	36	23.2
	19–20 years	38	24.5
	20–21 years	57	36.8
	21–22 years	8	5.1
	Over 22	10	6.5
Grade	Year 1	48	31
	Year 2	45	29
	Year 3	62	40
Home Region	Central China	59	38
	South China	16	10.3
	East China	39	25.1
	North China	14	9
	Northeast China	8	5.1
	Northwest China	9	5.8
	Southwest China	12	7.7

**Fig. 4:**
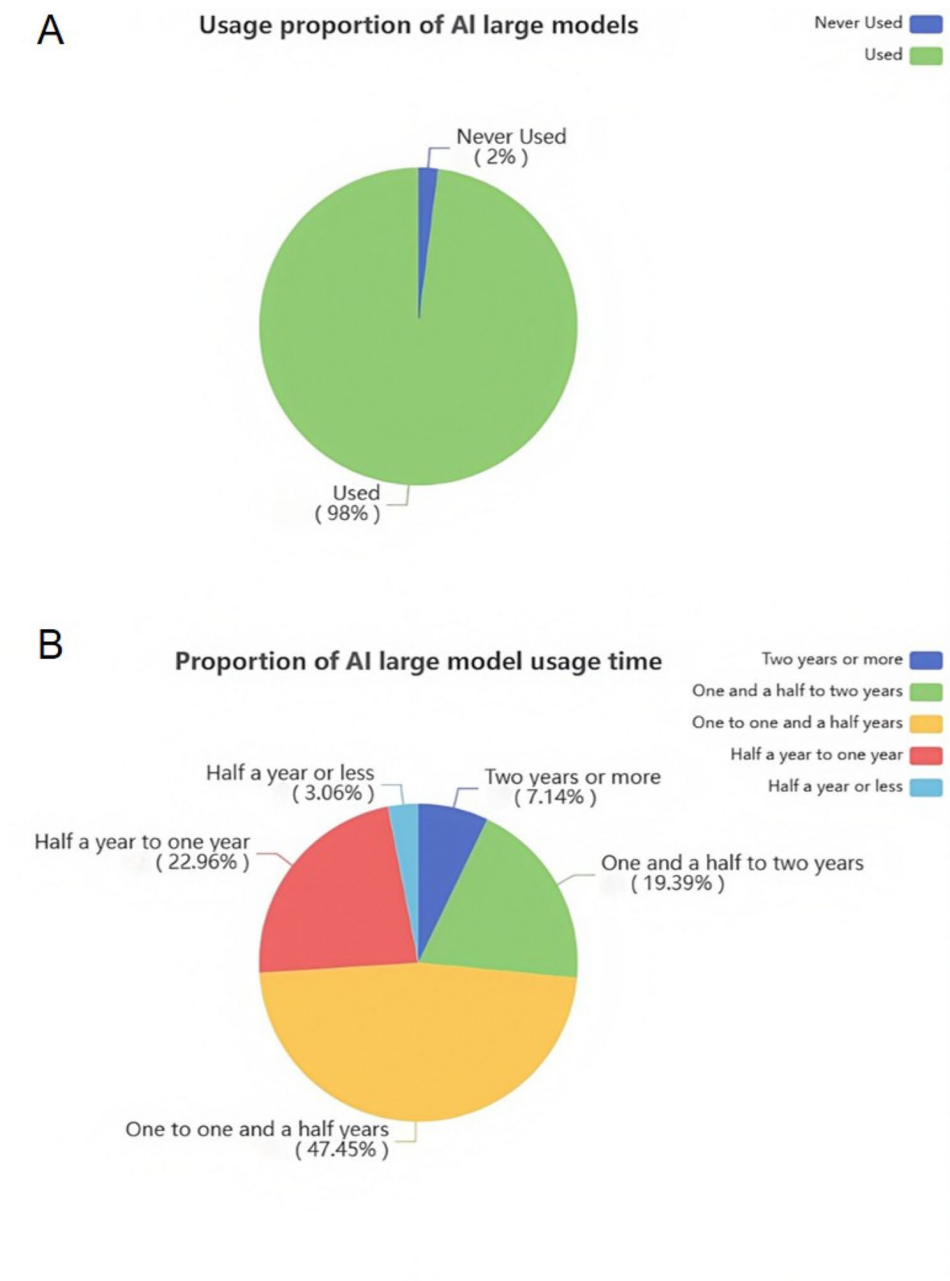
Participants' prior experience with AI large models. **A** The usage proportion of AI large models. **B** The proportion of AI large model usage time among users

To contextualize participants’ technological familiarity, the survey also assessed prior experience with large language models (LLMs), including ChatGPT, Gemini, DeepSeek, and Doubao. As shown in Fig. 4, digital literacy within the cohort was notably high: 98% (*n* = 152) of participants reported having prior experience with at least one LLM. Among these users, a substantial proportion (47.45%) had been using such tools for more than one year. It is important to note, however, that none of the participants had prior exposure to the custom-designed AI educational agent used in this study. Their evaluations thus reflect first-time interaction with the system.

Participant recruitment was conducted via official course communication groups between May and June, 2025. The recruitment notices informed students that the study aimed to investigate user experiences with an AI-based medical education platform and emphasized that participation was entirely voluntary. Participants first accessed the AI agent platform, where they were required to watch a 20-minute instructional video, followed by interactive tasks with a virtual teaching assistant and a virtual patient. Upon completion of the AI-based activities, participants were automatically redirected to the Wenjuanxing (Questionnaire Star) platform to complete the survey. After data screening, a final sample of 155 valid questionnaires was retained for subsequent analysis.

### Measures

The questionnaire items are detailed in Supplementary Table 2. The survey consisted of 28 items, all measured on a 5-point Likert scale (1 = Strongly Disagree, 5 = Strongly Agree). For the Perceived Risk construct, a higher score indicated a higher level of perceived risk. For instance, the item “If the medical agent gives erroneous feedback during critical operational steps, I will question its overall reliability” was used to measure the perception of performance risk.

### Data analysis strategy

This study employed Structural Equation Modeling (SEM) to test the proposed research model and hypotheses. Given the research objectives and data characteristics, Partial Least Squares (PLS) SEM was chosen as the analytical method, using SmartPLS 4.0 software. The choice of PLS-SEM was based on three main considerations. First, the study seeks to identify the relative importance of influencing factors in the emerging context of medical AI educational agents, which aligns with the predictive and exploratory orientation of PLS-SEM. Second, the research model is relatively complex, and PLS-SEM provides high robustness and explanatory power when dealing with intricate structural models. Third, the exploratory nature of this research makes PLS-SEM particularly appropriate, as it imposes fewer restrictions on both sample size and data distribution. Moreover, numerous empirical studies on behavioral intention have adopted PLS-SEM as their primary analytical method. For instance, Govind Lal et al. employed PLS-SEM to estimate and validate their model when investigating the antecedents of intention to use AI-powered transportation applications [42]. Similarly, Bilquise et al. (2024) applied PLS-SEM to examine students’ acceptance of academic advising chatbots in higher education institutions [43]. Additionally, methodological studies have demonstrated that PLS-SEM remains robust in modeling latent structures under conditions of non-normal data distribution and is particularly well-suited for small to medium-sized samples [44]. Therefore, it represents a methodologically sound and contextually appropriate analytical choice for this study.

The data analysis followed a two-stage approach: first, the measurement model was assessed for reliability and validity to ensure the quality of the survey data. Second, upon confirmation of the measurement model’s adequacy, the structural model was evaluated to test the significance of the path relationships and validate the research hypotheses. A bootstrapping procedure with 5,000 resamples was used to determine the significance levels (p-values) and t-statistics for the path coefficients.

## Results

### Sample characteristics

The final valid sample for this study consisted of 155 clinical medicine students from Tongji Medical College of Huazhong University of Science and Technology. Among them, there were 93 first- and second-year students and 62 third-year students.

### Measurement model evaluation

The measurement model was assessed for reliability and validity.

#### Reliability and convergent validity

Internal consistency reliability was examined using Cronbach’s Alpha and Composite Reliability (CR/rho_c). As shown in Table 2, all latent variables demonstrated good internal consistency, with Cronbach’s Alpha values exceeding 0.70 and CR values surpassing 0.80.

**Table 2:** Results of reliability and convergent validity tests

Construct	Code	Factor	Cronbach’s α	rho_c	AVE
PE	PE1	0.960	0.881	0.927	0.809
	PE2	0.899			
	PE3	0.835			
EE	EE1	0.801	0.895	0.922	0.704
	EE2	0.846			
	EE3	0.819			
	EE4	0.860			
	EE5	0.866			
SI	SI1	0.888	0.864	0.917	0.787
	SI2	0.886			
	SI3	0.886			
FC	FC1	0.846	0.887	0.922	0.747
	FC2	0.866			
	FC3	0.856			
	FC4	0.889			
PR	PR1	0.873	0.846	0.907	0.765
	PR2	0.853			
	PR3	0.898			
HM	HM1	0.911	0.758	0.892	0.804
	HM2	0.883			
AIT	AIT1	0.885	0.871	0.921	0.795
	AIT2	0.929			
	AIT3	0.860			
BI	BI1	0.866	0.869	0.920	0.793
	BI2	0.913			
	BI3	0.891			
TR	TR1	0.930	0.847	0.929	0.867
	TR3	0.933			

Convergent validity was evaluated using factor loadings and the Average Variance Extracted (AVE). The standardized factor loadings for all items ranged from 0.801 to 0.960, well above the recommended threshold of 0.70. Furthermore, as presented in Table 2, the AVE values for all latent variables ranged from 0.704 to 0.867, exceeding the recommended value of 0.50. These results provide strong evidence for the convergent validity of the measurement model.

#### Discriminant validity

Discriminant validity was assessed using the Fornell-Larcker criterion [45], which requires the square root of a construct’s AVE to be greater than its correlation coefficients with all other constructs. The results, displayed in Table 3, show that this condition was met for all constructs, confirming that the measures are distinct and the model has good discriminant validity.

**Table 3:** Fornell-Larcker criterion test results

	AIT	HM	FC	EE	TR	PR	SI	PE	BI
AIT	**0.892**								
HM	0.792	**0.897**							
FC	0.866	0.838	**0.864**						
EE	0.883	0.818	0.897	**0.839**					
TR	0.836	0.799	0.841	0.881	**0.931**				
PR	0.832	0.779	0.867	0.873	0.843	**0.875**			
SI	0.845	0.802	0.875	0.886	0.861	0.867	**0.887**		
PE	0.872	0.845	0.912	0.915	0.870	0.879	0.894	**0.900**	
BI	0.847	0.785	0.887	0.899	0.834	0.849	0.850	0.898	**0.890**

(Note: In Table 3, the bold values on the diagonal represent the square root of the AVE.)

### Structural model evaluation and hypothesis testing

After establishing the reliability and validity of the measurement model, the structural model was evaluated.

#### Model explanatory power and fit

The model’s goodness-of-fit was assessed using the Standardized Root Mean Square Residual (SRMR). The SRMR value for this study was 0.099, which is close to the acceptable threshold of 0.10, indicating an acceptable fit between the model and the data.

The explanatory power of the model was measured by the coefficient of determination (R²). The model explained 66.9% of the variance in Effort Expectancy (R² = 0.669), 87.4% of the variance in Performance Expectancy (R² = 0.874), and 85.3% of the variance in the final dependent variable, Behavioral Intention (R² = 0.853). According to the criteria proposed by Falk and Miller (1992), R² values above 0.10 are considered substantial. The R² values in this model far exceed this standard, indicating very strong predictive and explanatory power.

#### Hypothesis testing

**Fig. 5:**
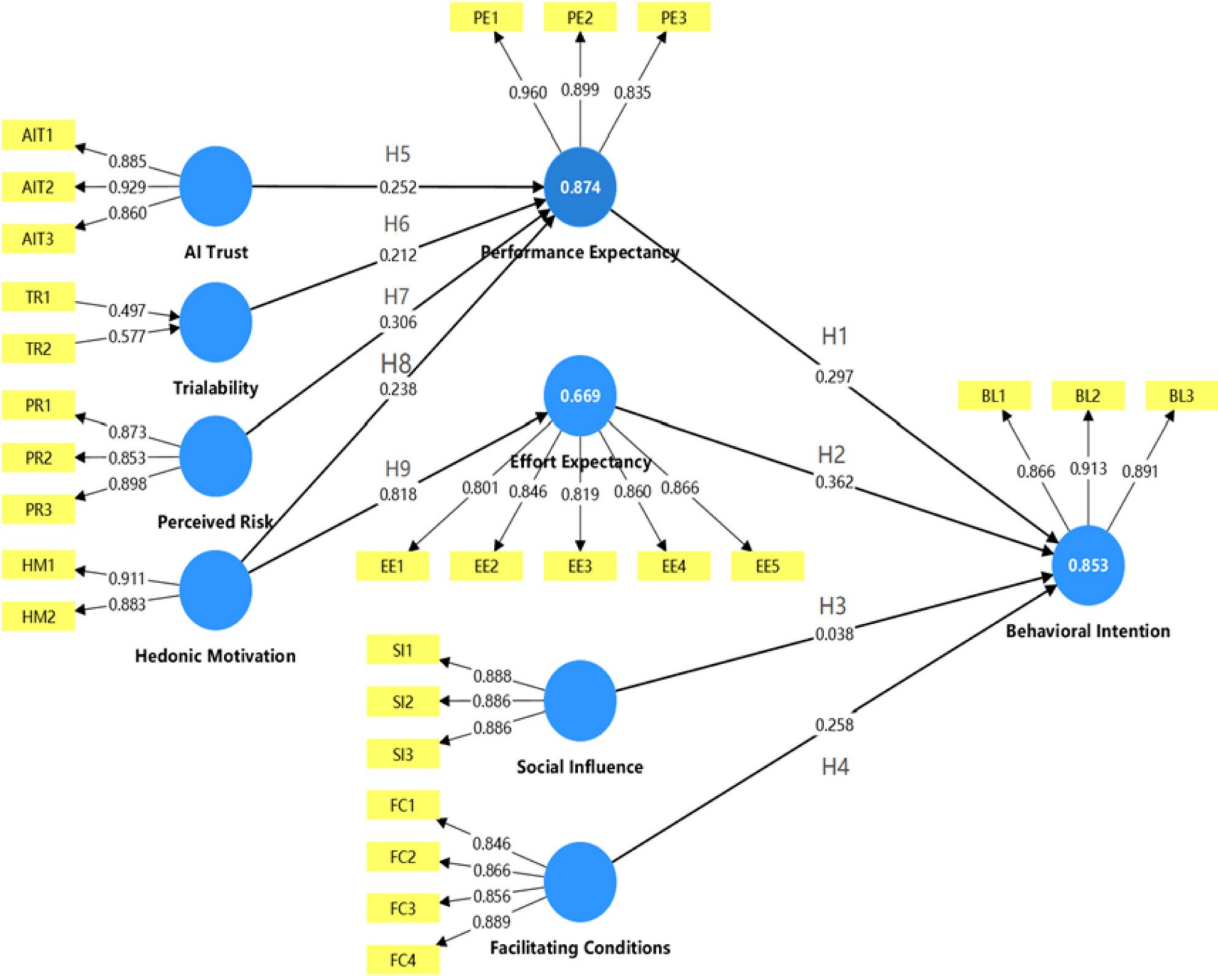
Structural model hypothesis testing. Note: In Fig. 5, the numbers on the thin arrows represent indicator loadings, while the numbers on the thick arrows represent path coefficients. H1-H9 denote Hypotheses 1 through 9

The research hypotheses were tested using a bootstrapping procedure (5,000 resamples). The results are presented in Table 4; Fig. 5.

**Table 4:** Structural model path coefficients and hypothesis testing results

Hypothesis	Path Relationship	Path Coefficient (β)	TStatistics	*P*-value	Supported
H1	Performance Expectancy ->Behavioral Intention	0.297	3.416	< 0.001	Yes
H2	Effort Expectancy ->Behavioral Intention	0.362	4.499	< 0.001	Yes
H3	Social Influence ->Behavioral Intention	0.038	0.477	0.633	No
H4	Facilitating Conditions ->Behavioral Intention	0.258	3.069	0.002	Yes
H5	AI Trust ->Performance Expectancy	0.254	4.071	< 0.001	Yes
H6	Trialability ->Performance Expectancy	0.210	3.424	< 0.001	Yes
H7	Perceived Risk ->Performance Expectancy	0.306	4.568	< 0.001	Yes
H8	Hedonic Motivation ->Performance Expectancy	0.237	4.155	< 0.001	Yes
H9	Hedonic Motivation ->Effort Expectancy	0.818	29.754	< 0.001	Yes

Of the nine hypothesized paths, eight were supported, and one was not. The path from Social Influence to Behavioral Intention was not significant (β = 0.038, *p* = 0.633), and therefore, H3 was not supported. All other hypothesized paths had p-values less than 0.05, indicating strong statistical support from the data. Specifically:

Antecedents of Performance Expectancy: AI Trust (β = 0.254, *p* < 0.001), Trialability (β = 0.210, *p* < 0.001), and Hedonic Motivation (β = 0.237, *p* < 0.001) all had significant positive effects on Performance Expectancy, supporting H5, H6, and H8. H7 predicted a negative relationship between Perceived Risk and Performance Expectancy. The path coefficient was β = 0.306 (*p* < 0.001). Since the scale was coded such that higher scores indicate higher perceived risk, this positive coefficient statistically confirms that higher risk is associated with lower performance expectancy, thus supporting H7.

Antecedent of Effort Expectancy: Hedonic Motivation had a very strong positive effect on Effort Expectancy (β = 0.818, *p* < 0.001), supporting H9.

Antecedents of Behavioral Intention: Performance Expectancy (β = 0.297, *p* < 0.001), Effort Expectancy (β = 0.362, *p* < 0.001), and Facilitating Conditions (β = 0.258, *p* = 0.002) all had significant positive effects on students’ behavioral intention to use the medical AI agent, supporting H1, H2, and H4, respectively.

## Discussion

### Discussion of key research findings

#### Utility and usability as cornerstones: validation of core UTAUT variables

This study confirms that three core constructs of the UTAUT model—Performance Expectancy (PE), Effort Expectancy (EE), and Facilitating Conditions (FC)—all have a significant positive impact on medical students’ behavioral intention (BI). Effort Expectancy (β = 0.362) and Performance Expectancy (β = 0.297) emerged as the most powerful direct drivers of BI. This not only validates the applicability of the UTAUT model in the medical education context but also reveals a fundamental truth: for medical students facing immense academic and clinical pressures, the adoption of any new tool is predicated on a rational cost-benefit analysis. When they perceive that an AI agent can deliver tangible learning gains (e.g., efficiently understanding complex concepts, improving diagnostic skills) and that its use is seamless without imposing additional cognitive load, the intention to adopt naturally follows.

Furthermore, the significant influence of Facilitating Conditions (β = 0.258) highlights the importance of “ecosystem” support, underscoring that individual intention requires organizational resources to be realized. This suggests that even if students are willing to use an agent, this intention may not translate into action if they lack necessary hardware (e.g., computers, stable internet) and software support (e.g., clear instructions, timely technical help). This finding is highly consistent with the results of Chen et al.‘s study on “Rain Classroom” and the conclusions of most technology acceptance models [40, 41, 45].

#### The power of experience: the potent driving force of hedonic motivation

A crucial finding of this study is the powerful role of Hedonic Motivation (HM). HM not only significantly influenced Performance Expectancy (β = 0.237,*p* < 0.001) but also demonstrated an exceptionally strong predictive power on Effort Expectancy (β = 0.818). This reveals a profound mechanism: the value of an engaging and immersive learning tool extends far beyond mere “fun.” When the learning process itself is captivating (e.g., interacting with a virtual patient), students subjectively perceive the tool as being less complex and “effortless” to use, which dramatically increases their Effort Expectancy. Simultaneously, this highly engaging, contextualized learning experience reinforces their belief that they can acquire genuine knowledge and skills, thereby boosting their Performance Expectancy. The practical implications of this finding are substantial: for developers, investing in user experience and enjoyment is not a superficial addition but a critical factor that directly impacts core acceptance variables (EE and PE).

#### Trust and safety as prerequisites: the specificity of medical education

The results show that higher AI Trust (β = 0.254, *p* < 0.001) leads to higher Performance Expectancy. In this study, Perceived Risk was measured using positively framed items, where a higher score indicates lower perceived risk and higher system safety. Therefore, the positive path coefficient (β = 0.306, *p* < 0.001) indicates that lower perceived risk corresponds to higher Performance Expectancy. This finding underscore that in the medical domain, a tool’s “usefulness” (PE) is not determined by its functionality alone but is anchored by trust. If students doubt the accuracy or authority of the information provided by the AI or worry about potential negative consequences (such as developing flawed clinical reasoning), they will not perceive the tool as genuinely helpful, regardless of how powerful its features are. This emphasizes that establishing trust mechanisms (e.g., clearly citing information sources, providing evidence-based rationale, explaining the reasoning process) is a fundamental prerequisite for the acceptance of AI educational tools in healthcare [46].

Trialability (TR) also had a significant positive effect on Performance Expectancy (β = 0.210), which is consistent with Rogers’ (2003) Diffusion of Innovations Theory [19]. For a complex new tool, allowing users to experiment and explore in a low-risk environment is an effective way to build their perception of its value and their confidence in using it.

#### The “Failure” of social influence: a manifestation of professional learner autonomy

Contrary to many traditional UTAUT studies, the effect of Social Influence (SI) on Behavioral Intention (BI) was not significant in this research (β = 0.038, *p* = 0.633). We contend that this is not merely a null result, but rather a meaningful finding that underscores the unique characteristics of our sample. Medical students, as advanced adult learners and emerging professionals, tend to exhibit a high degree of autonomy in their educational decision-making. Their willingness to adopt new technology appears to be driven primarily by personal, rational evaluations of its value—such as its perceived ability to enhance academic performance (Performance Expectancy) or the enjoyment derived from its use (Hedonic Motivation)—rather than by peer pressure or external social expectations.

This finding delineates an important contextual boundary for the application of the UTAUT model. While Social Influence may be a strong predictor in mandatory or more generalized educational environments, its explanatory power appears to diminish among learners who possess high levels of intrinsic motivation and professional self-direction. In highlighting this limitation, our study contributes a nuanced perspective to the UTAUT framework, identifying the specific conditions under which one of its core constructs becomes less salient.

The practical implication for medical education administrators and technology promoters is clear: traditional promotional strategies that rely on endorsements from authority figures or peer conformity may have limited effectiveness in this context. Instead, efforts should focus on clearly articulating and demonstrating the individual value of the technology to students in terms of both academic utility and user experience.

### Theoretical and practical significance

#### Theoretical implications

This study successfully applies and extends the UTAUT model to the novel and unique domain of “AI medical education agents.” More importantly, it reveals how the core utilitarian attributes of technology (PE, EE) are co-shaped by users’ psychological perceptions (trust, risk), subjective experiences (hedonic motivation), and behavioral processes (trialability) in a high-risk, high-professionalism educational context. This provides new empirical support and a theoretical lens for the advancement of technology acceptance theories from general models to specialized professional domains.

Furthermore, this research deepens the understanding of the boundary conditions for the social influence construct. The finding that social Influence is not significant provides an important boundary condition for UTAUT, suggesting that user professionalism, learning autonomy, and the risk level of the decision-making context may be key variables moderating the strength of its effect. Future technology acceptance research should consider the heterogeneity of user groups rather than assuming the universal applicability of social influence.

#### Practical implications

The results of this study offer significant practical guidance for medical students, AI agent developers, and educational administrators.

For Medical Students: This study advocates for students to become “prudent drivers of AI.” They should view AI agents as powerful assistants, not as infallible authorities. While leveraging their convenience, students must maintain critical thinking, actively verify information sources, and use their interactions with AI as a training ground for developing evidence-based reasoning skills. Students should proactively use the personalized and engaging features of AI to target their specific areas of weakness, consciously internalizing AI from an external tool into an integral part of their personal knowledge system to achieve highly efficient and deeply personalized learning.

For AI Educational Agent Developers: The findings suggest a three-tiered strategy of “usable, trustworthy, and engaging.” Given the critical importance of Effort Expectancy, making the agent “usable” by simplifying operations, optimizing interactions, and enhancing system responsiveness must be the top priority. To make it “trustworthy,” developers must address the core concerns of AI Trust and Perceived Risk by providing clear evidence-based citations for AI-generated knowledge and establishing transparent feedback and correction mechanisms. Finally, to make it “engaging,” developers should leverage the powerful influence of Hedonic Motivation by incorporating gamification, high-quality 3D models, and realistic virtual patients to transform learning into an attractive exploratory experience, thereby increasing user retention.

For Hospital and Education Administrators: The significant role of Facilitating Conditions suggests that administrators should transition from being “tool distributors” to “ecosystem builders.” This includes providing stable networks, high-performance equipment, comprehensive user guides, and accessible technical support channels to clear all external barriers to adoption. The weak effect of Social Influence serves as a reminder that top-down administrative mandates or simple word-of-mouth marketing may be ineffective. A more potent strategy is to focus on demonstrating value, organizing case-sharing sessions and showcasing learning outcomes to let students see firsthand how the tool helps their peers solve real learning challenges, thus intrinsically motivating adoption.

### Limitations and future research

This study has several limitations that warrant further investigation in future research:

Sample Limitations: The sample was drawn from a single medical college and consisted mainly of undergraduate students, which limits the generalizability of the findings to other student populations (e.g., postgraduate students, residents) and institutions. Future research should include a more diverse sample from multiple institutions and training levels.

Furthermore, regarding the sample size, while our valid sample (*N* = 155) meets the minimum recommended threshold for Structural Equation Modeling (SEM) as proposed by several methodological scholars [47], we fully acknowledge that this remains a relatively modest sample. Accordingly, we position this study as an exploratory, preliminary investigation into the emerging domain of AI educational agents in medical education.

The limited scope of our current sample is primarily due to the practical constraints of technological dissemination. While general-purpose large language models have become widespread, domain-specific AI agents tailored for medical education are still in the early stages of implementation and adoption. Our selection of Tongji Medical College, Huazhong University of Science and Technology, was a deliberate and strategic choice: as a top-tier institution and an early adopter of educational AI technologies, it provided a unique opportunity to study student interactions with an advanced system not yet widely available elsewhere. Nonetheless, we clearly recognize this as a limitation. As this technology matures and is adopted more broadly, we plan to extend our research to include students from a wider range of institutions across different tiers of the medical education system in China. This future expansion will allow us to capture a more diverse and representative sample, thereby improving the generalizability and external validity of our findings.

A significant limitation of this study lies in the potential influence of a novelty effect, which warrants a nuanced and contextualized discussion. This effect stems not merely from exposure to a new technology, but from a sharp contrast between the research tool and participants established technological frame of reference. As described in "Data Collection and Sample" section, nearly all participants (98%) were already familiar with general-purpose AI tools such as ChatGPT and DeepSeek, primarily through disembodied, text-based interfaces. In contrast, our AI agent introduced two notable innovations: embodiment (i.e., interaction with a visual persona representing a virtual teacher or patient) and multimodal communication (i.e., the ability to converse using both text and voice).

This departure from their habitual AI use likely heightened cognitive engagement and elicited a strong sense of novelty, which in turn may explain the exceptionally strong predictive effect of Hedonic Motivation (HM) in our results. The enjoyment and interest reflected in HM were not merely generic user responses, but were likely amplified by the novelty of interacting with a more human-like, immersive AI system. This interpretation positions the novelty effect not as a confounding factor to be minimized, but as a key explanatory lens for understanding the elevated role of hedonic factors in initial technology adoption.

However, it is important to note that this novelty-driven engagement is inherently transient. As students become more accustomed to the system, the initial “wow factor” is likely to diminish, potentially shifting their evaluation from emotional appeal toward more utilitarian considerations. Our current cross-sectional design captures only this early-stage interaction, and is thus limited in its ability to explore how motivational factors evolve over time. We therefore recommend that future research adopt a longitudinal design, tracking users over the course of a semester or academic year. This would allow for a more comprehensive understanding of how hedonic drivers may give way to performance-based or outcome-focused predictors in determining sustained use of educational AI technologies.

Variable Selection Limitations: Although the model has high explanatory power, other important variables, such as students’ personal innovativeness or differences in needs across clinical specialties, may not have been included. Future studies could incorporate additional moderating variables to explore more complex interactions.

This study employed a specific large language model (DeepSeek-R1) as the foundation for the AI educational agent. Given the rapid evolution of generative AI technologies, the capabilities and interaction quality of different models can significantly influence user perceptions and acceptance. As such, comparative research that examines how variations in underlying AI architectures affect student adoption and learning outcomes would be a valuable direction for future investigation.

Another technological limitation pertains to the issue of timeliness, stemming from the fast-paced nature of AI development. The medical educational agent used in this study was primarily developed in 2024, with its technical architecture and model capabilities reflecting the state-of-the-art at that time. However, the actual implementation and data collection were carried out in 2025. Due to the swift advancements in large language models and AI infrastructure, the system used in this research may no longer represent the most current technological frontier. This temporal gap raises important questions about the durability and generalizability of our findings in light of newer, more advanced AI capabilities.

To address this challenge and ensure the agent’s continued relevance, our development team has initiated ongoing updates and iterative improvements. For example, we have recently integrated an emotion recognition module that enables the AI agent to better perceive and respond to students’ real-time emotional and cognitive states during interaction. This upgrade not only helps mitigate the temporal limitations of the version used in this study but also opens promising new research avenues. In particular, it paves the way for future work exploring how context-aware and affect-sensitive medical AI educational agents may influence student engagement, learning outcomes, and sustained adoption in medical education settings.

## Conclusion

This study employed an extended UTAUT model to investigate the factors influencing medical students’ intention to adopt AI educational agents. The model demonstrated strong explanatory power (85.3%) and identified three key drivers: Performance Expectancy, Effort Expectancy, and Hedonic Motivation. These findings suggest that adoption is driven by a pragmatic evaluation of utility and enjoyment, rather than by external social influence. A notable theoretical contribution is the identification of a boundary condition for Social Influence, which proved non-significant in this context. This highlights the importance of user autonomy in professional education settings and suggests that traditional influence-based strategies may have limited impact. Practically, the results offer clear guidance for system developers and educators: prioritize usability, foster trust, and design engaging learning experiences. Adoption efforts should focus on demonstrating individual value to students, rather than relying on top-down persuasion. Ultimately, successful integration of AI in medical education depends not only on technological innovation, but on alignment with the needs, values, and autonomy of learners themselves.

## Supplementary Information

Supplementary Material 1

Supplementary Material 2

Supplementary Material 3

Supplementary Material 4

## Data Availability

The anonymized questionnaire response data used in this study are provided in the supplementary material (Supplementary Table 3). A demo version of the custom-developed medical education AI agent is also included as Supplementary Material 4 (medical AI educational agents for medical student) to illustrate the system’s core functionalities and user interface. Additional materials, including raw statistical output files and system architecture details, are available from the corresponding author upon reasonable request.

## Abbreviations

TAM: Technology Acceptance Model
AIT: AI Trust
HM: Hedonic Motivation
TR: Trialability
PR: Perceived Risk
PE: Performance Expectancy
EE: Effort Expectancy
SI: Social Influence
FC: Facilitating Conditions
LLM: Large Language Model
HM: Hedonic Motivation
