# Supplementary material for: Factors influencing medical students’ adoption of AI educational agents: an extended UTAUT model
Source: BMC Med Educ. 2025 Dec 5;25:1678. doi: 10.1186/s12909-025-08234-z (PMC12681116; doi:10.1186/s12909-025-08234-z)
Supplement: Supplementary file 1 — Supplementary Material 1 [file 12909_2025_8234_MOESM1_ESM.docx]

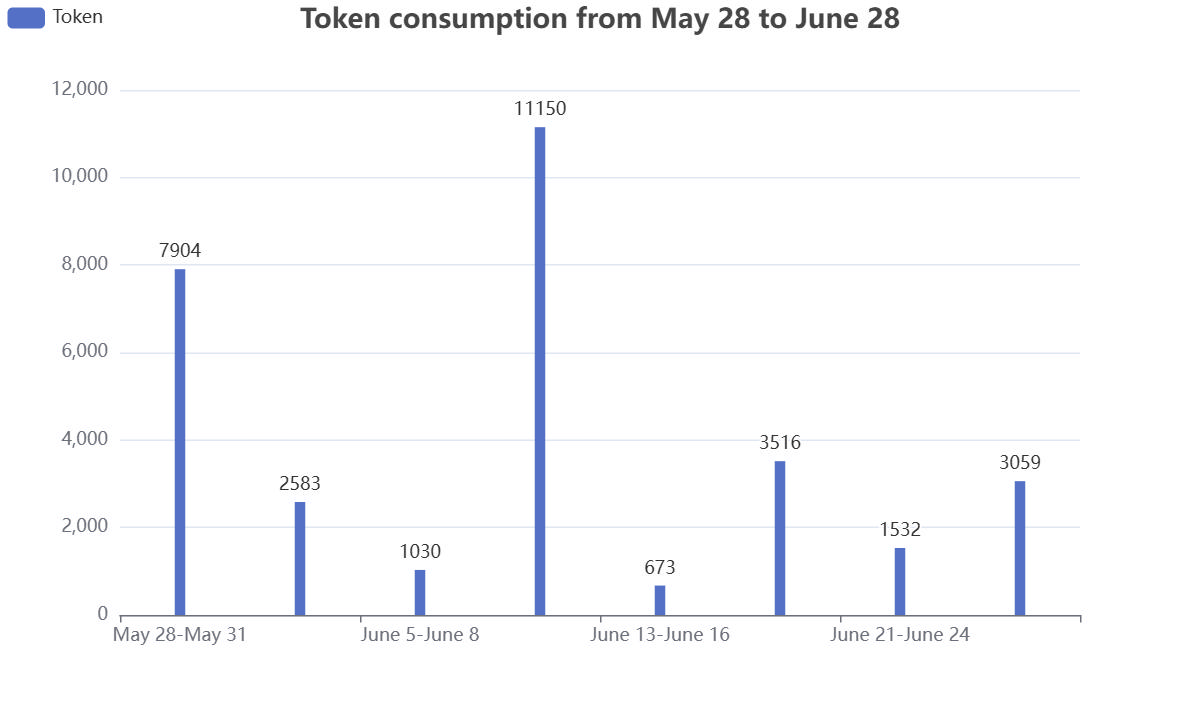


Figure1 Daily Token Consumption During the Study Period


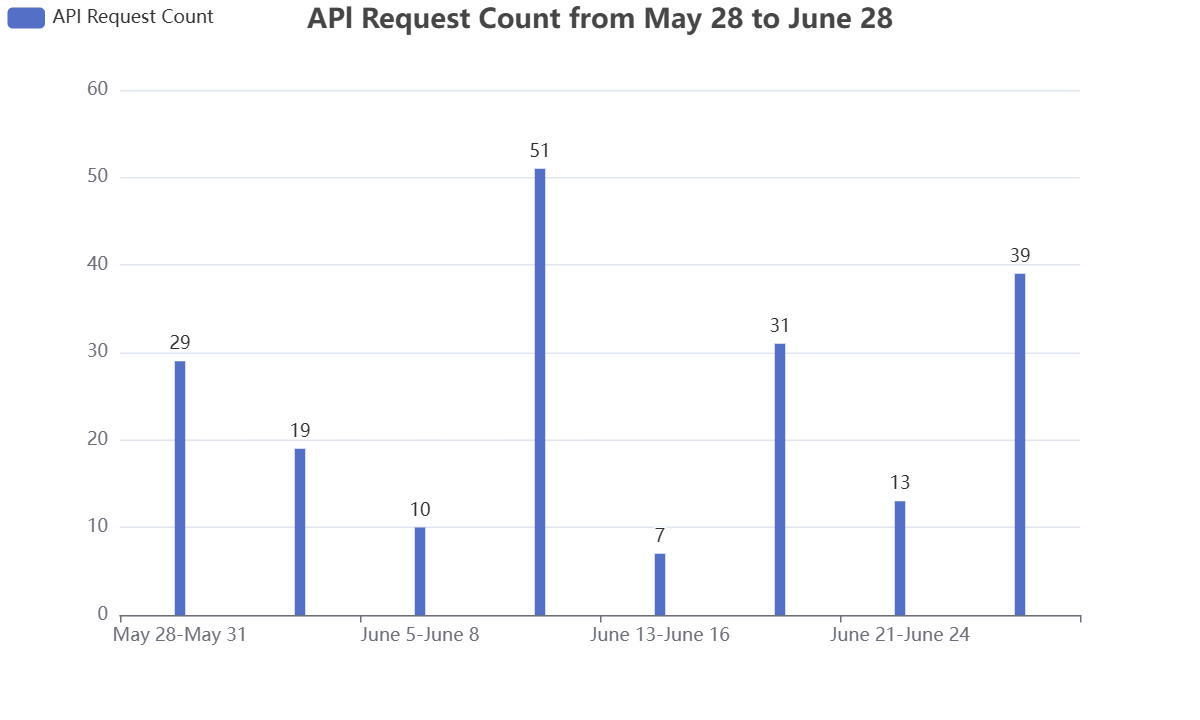


Figure 2 Daily API Request Count During the Study Period


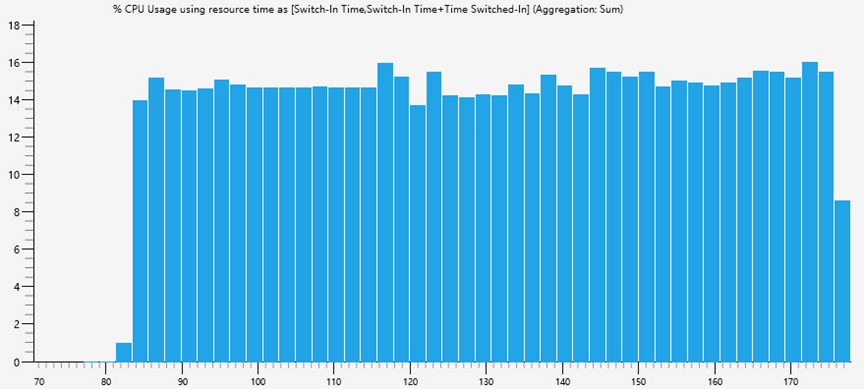


Figure 3 CPU Usage Percentage During Agent Operation
