# Supplementary material for: Factors influencing medical students’ adoption of AI educational agents: an extended UTAUT model
Source: BMC Med Educ. 2025 Dec 5;25:1678. doi: 10.1186/s12909-025-08234-z (PMC12681116; doi:10.1186/s12909-025-08234-z)
Supplement: Supplementary file 2 — Supplementary Material 2 [file 12909_2025_8234_MOESM2_ESM.docx]

| **Question Design** | **Code** | **Measurement Item** | **Reference Sources** |
| --- | --- | --- | --- |
| Performance Expectancy (PE) | PE1 | The medical agent helps me achieve my learning goals more efficiently. | Venkatesh et al. (2003);  DeLone & McLean (2003); Alqaisi N. et al. (2025);  Granić A. (2023) |
|  | PE2 | Using the medical agent improves my learning outcomes. |  |
|  | PE3 | Using the multi-modal interaction features of the medical agent (e.g., 3D models, animations) helps me better understand complex knowledge points. |  |
| Effort  Expectancy (EE) | EE1 | The operational interface layout of the medical agent is clear and reasonable; function entry points are easy to locate. | Venkatesh et al. (2003);  Davis F.D. (1989);  Zhai H et al. (2021);  Hunde MK. et al. (2023);  Teo T et al. (2023) |
|  | EE2 | I believe it does not require much effort to learn the main functions of the medical agent platform. |  |
|  | EE3 | The currently used medical agent supports voice/text interaction, allowing me to use it without learning complex operations. |  |
|  | EE4 | The medical agent can respond quickly to my questions or other operations. |  |
|  | EE5 | During conversations, the medical agent can understand complex questions and provide logically coherent answers. |  |
| Social  Influence (SI) | SI1 | In daily life, classmates or friends recommend that I use the medical agent. | Venkatesh et al. (2003);  Rogers E. M. (2003);  Yakubu MN et al. (2025);  Su J et al. (2025) |
|  | SI2 | Teachers and the school believe that using the medical agent is helpful for learning. |  |
|  | SI3 | Industry trends (e.g., academic conferences, media reports) make me feel that using medical agents is the direction of future development. |  |
| Facilitating Conditions (FC) | FC1 | I have sufficient devices (e.g., computer, phone) and stable internet to support my use of the medical agent. | Venkatesh et al. (2003);  Chatterjee S. et al. (2023);  Sichone J et al. (2018) |
|  | FC2 | When I encounter problems using the medical agent, I know how or where to seek help (e.g., clear documentation or technical support channels). |  |
|  | FC3 | I believe I possess the necessary knowledge and skills to smoothly use the medical agent. |  |
|  | FC4 | The hospital provides us with the necessary guidance or training for using the medical agent. |  |
| Perceived  Risk (PR) | PR1 | If the medical agent gives erroneous feedback during critical operational steps, I will question its overall reliability. | Bauer RA (1960);  Shin D (2021);  Ramot S&Tal O. (2024);  Deng Z et al. (2018)  Teng Z. et al. (2022);  Slade EL. et al. (2015) |
|  | PR2 | If the AI's explanations for disease diagnosis and treatment recommendations are contradictory or erroneous (e.g., recommending contraindicated medications) and there is no clear error-correction mechanism, I will cautiously adopt its subsequent advice. |  |
|  | PR3 | If the diagnostic and treatment suggestions provided by the AI do not specify clear data sources or evidence-based references, my trust in that advice will decrease. |  |
| Hedonic Motivation (HM) | HM1 | The process of performing operational training in a simulated environment is inherently attractive and fun. | Van der Heijden (2004);  Teng Z et al. (2022);  Wang L&Li W. (2024) |
|  | HM2 | Interacting with highly realistic virtual patients helps me practice clinical decision-making processes (e.g., consultation, differential diagnosis) in an immersive way. |  |
| AI Trust (AIT) | AIT1 | I trust that the medical knowledge provided by the medical agent (especially the AI large model component) is accurate and up-to-date. | Lee JD, See KA. (2004);  Gefen D.et al. (2003); Lee J-H,&Song C-H.(2013);  Teng Z. et al. (2022) |
|  | AIT2 | I believe virtual patients can realistically simulate disease states, and their feedback is reliable. |  |
|  | AIT3 | I trust that the design purpose of this agent is to enhance my clinical skills, and its operation process is benevolent and reliable. |  |
| Behavioral Intention (BI) | BI1 | I am willing to continue using the medical agent in the future. | Davis F.D. (1989); Bandura (1986); Venkatesh et al. (2003) |
|  | BI2 | I am willing to recommend the medical agent to others. |  |
|  | BI3 | I plan to frequently use the medical agent in my upcoming studies and work. |  |
| Trialability (TR) | TR1 | Before formal implementation, I have the opportunity to trial the core functions of the medical agent (e.g., interacting with virtual patients, using AI for Q&A). | Rogers, E. M. (2003);  Chaibi A.et al. (2025);  Lee J-H, Song C-H. (2013);  Teng Z. et al. (2022) |
|  | TR3 | I can fully understand the role of this medical agent through the instructional demos or trial modules provided by the platform. |  |
